# The SCLtTAxBCR-ABL transgenic mouse model closely reflects the differential effects of dasatinib on normal and malignant hematopoiesis in chronic phase-CML patients

**DOI:** 10.18632/oncotarget.16152

**Published:** 2017-03-13

**Authors:** Claudia Schubert, Nicolas Chatain, Till Braunschweig, Mirle Schemionek, Kristina Feldberg, Melanie Hoffmann, Olli Dufva, Satu Mustjoki, Tim H. Brümmendorf, Steffen Koschmieder

**Affiliations:** ^1^ Department of Hematology, Oncology, Hemostaseology, and Stem Cell Transplantation, Faculty of Medicine, RWTH Aachen University, Aachen, Germany; ^2^ Institute of Pathology, Faculty of Medicine, RWTH Aachen University, Aachen, Germany; ^3^ Hematology Research Unit Helsinki, Department of Clinical Chemistry and Hematology, University of Helsinki and Helsinki University Hospital Comprehensive Cancer Center, Helsinki, Finland

**Keywords:** CML, BCR-ABL, dasatinib, mouse model, transgenic

## Abstract

The second generation tyrosine kinase inhibitor (TKI) dasatinib is a clinically approved drug for chronic myeloid leukemia (CML) as well as Ph^+^ acute lymphoblastic leukemia. In addition to its antileukemic effects, dasatinib was shown to impact on normal hematopoiesis and cells of the immune system.

Due to the fact that the murine *in vivo* studies so far have not been performed in a chronic-phase CML model under steady-state conditions, our aim was to study the hematopoietic effects of dasatinib (20 mg/kg p.o.) in BCR-ABL expressing SCLtTAxBCR-ABL double transgenic (dtg) mice. Dasatinib robustly antagonized the CML phenotype *in vivo* in our transgenic mouse model, and this effect included both mature and immature cell populations. However, similar to patients with CML, the fraction of Lin^neg^Sca-1^+^KIT^+^CD48^neg^CD150^+^ hematopoietic stem cells was not reduced by dasatinib treatment, suggesting that these cells are not oncogene-addicted. Moreover, we observed differential effects of dasatinib in these animals as compared to wild-type (wt) animals: while granulocytes were significantly reduced in dtg animals, they were increased in wt mice. And Ter119^+^ erythrocytic and B220^+^ B cells were increased in dtg mice but decreased in wt mice. Finally, while dasatinib induced a shift from CD49b/NK1.1 positive NK cells from the bone marrow to the spleen in wt animals, there was no change in dtg mice. In conclusion, the present mouse model provides a useful tool to study mechanisms of TKI resistance and dasatinib-associated beneficial effects and adverse events.

## INTRODUCTION

Dasatinib is a second-generation ABL tyrosine kinase inhibitor (TKI), approved for the treatment of chronic myeloid leukemia (CML) and BCR-ABL-positive acute lymphoblastic leukemia (ALL). However, due to its multitarget specificity, it is also being tested in clinical trials for other cancers. Dasatinib has been shown to exert marked effects on the normal hematopoietic system [1–3]. *In vitro*, dasatinib induces immunosuppression by targeting key kinases of the immune system such as Lyn and Brutons's tyrosine kinase (Btk), thereby inhibiting B- and T cell signaling [4]. Furthermore, several studies showed an immunomodulatory effect of dasatinb, including mobilization of natural killer (NK) cells and enhanced NK cell cytotoxicity, both of which were not seen with imatinib or nilotinib [5–8]. The immunomodulatory function of dasatinib and the activation of large granular lymphocytes (LGLs) were shown to be associated with a better outcome, being associated with increased cytogenetic and molecular remissions [7, 9]. A correlation between LGL lymphocytosis and the development of autoimmune symptoms such as pleural effusions and colitis has been postulated [10]. Pleural effusions were more likely induced by accumulation of lymphocytes than by fluid retention alone [11].

Several studies have addressed the influence of dasatinib on non-malignant hematopoietic cells. Schade et al and Blake et al demonstrated that dasatinib inhibits T cell receptor (TCR) signal transduction, resulting in reduced proliferation and cytokine production but not apoptosis of T cells, if dasatinib is constantly present [12, 13]. Conversely, dasatinib induced apoptosis in human B-lymphocytes derived from peripheral blood or bone marrow, resulting in a strong reduction of B cells [2]. Through off-target inhibition of Btk, this reduction also resulted in reduced memory B cell numbers and impairment of the B cell response in dasatinib-treated patients [14]. Importantly, *in vitro* and *in vivo* data of dasatinib treatment can differ significantly in T cell but also NK cell differentiation and activation, due to the short half-life of the TKI in plasma in comparison to the endured presence in cell culture [15, 16].

We studied the hematopoietic effects of dasatinib in BCR-ABL expressing SCLtTAxBCR-ABL double transgenic (dtg) mice [17]. This model allows the analysis of dasatinib-induced effects on mature blood and immune cells as well as on hematopoietic stem and progenitor cells in BCR-ABL expressing and control mice. In wild-type mice, dasatinib had been shown to transiently activate hematopoietic stem cells (HSCs) and induce the proliferation of HSCs and a loss of lineage-committed progenitor cells, and this was associated with increases of stem cell factor (SCF) serum levels [18]. Given that our previous studies had demonstrated that BCR-ABL induces an increase of Lin^-^Sca-1^+^Kit^+^ (LSK) cells and granulocyte-macrophage progenitor cells (GMPs) [17], we now studied how dasatinib influences the proliferation and differentiation of hematopoietic stem and progenitor cells. Moreover, with our observation that BCR-ABL expression leads to a strong decrease of B220^+^ B cells in the bone marrow and the spleen [19], and with the evidence by Oksvold and colleagues shown that dasatinib induces apoptosis in normal B cells [2], we studied how dasatinib affects B- and T cells as well as NK cells in a CML *in vivo* model (both in a steady-state setting or after transplantation of BCR-ABL positive bone marrow stem cells). Finally, we studied whether dasatinib is able to reverse the BCR-ABL induced CML phenotype, including organ infiltration by myeloid cells, splenomegaly, and bone marrow stem and progenitor cell expansion.

## RESULTS

### Dasatinib-induced changes of the immunophenotype in wild-type mice

In this study, we evaluated the effect of dasatinib on different cell types in bone marrow (BM), spleen and peripheral blood (PB) by FACS analysis. An overview of the different mice and treatment schedules is depicted in Supplementary Figure 1.

**Figure 1:**
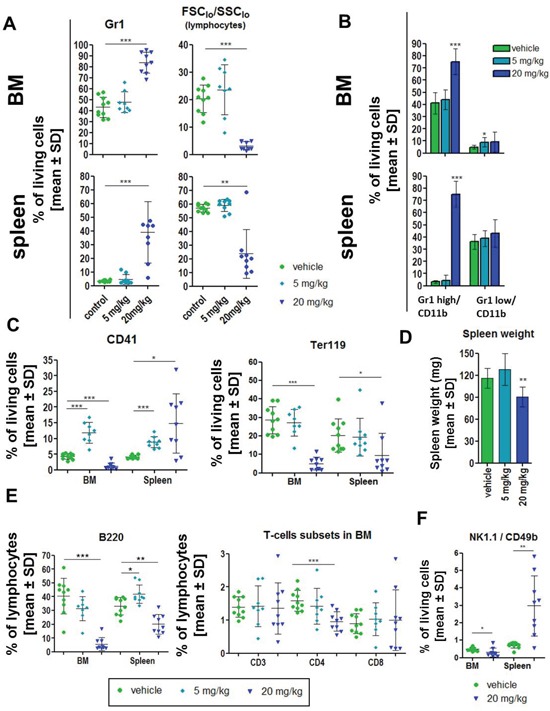
Dasatinib induced effects in wild-type FVB/N mice Wild-type animals were treated for 14 days with vehicle control (n = 10; green), 5 mg/kg (n = 8; light blue) or 20 mg/kg dasatinib (n = 9; dark blue) and the spleen and bone marrow (BM) cells were analyzed by FACS. **(A)** Percent of Gr1 positive granulocytes (gated on living cells), as well as percentage of lymphocytes (% gated on FCS_low_^/^SSC_low_). **(B)** Differentiation of mature (Gr1 high/CD11b expression) and immature (Gr1 low/CD11b expression) granulocytes in BM and spleen. **(C)** Evaluation of the percentage of megakaryocytes (CD41^+^) and erythroid cells (Ter119^+^) in BM and spleen gated on living cells. **(D)** Analysis of the spleen revealed a significant loss of spleen weight after 20 mg/kg dasatinib. **(E)** Detailed characterization of lymphocytes by staining for B220^+^ (B cells), CD3, CD4 and CD8 (T cells) in BM and spleen. **(F)** Natural killer cells (NK cells) stained for CD49b and NK1.1 double positivity in the BM and the spleen. All data are shown as mean ± SD. *p < 0.05.

We analyzed the immunophenotype in BM and spleen of 8 week old FVB/N wt mice treated for 14 days with 5 or 20 mg/kg dasatinib compared to vehicle control (5% DMSO in citrate buffer). The concentrations where based on previous *in vivo* experiments [12, 20]. The last dosing of dasatinib was performed on the day before the final analysis. Flow cytometry measurements revealed a significant increase of Gr1 positive granulocytes in the BM and spleen that was associated with a significant reduction of the fraction of lymphocytes (Figure 1A). This was due to a significant increase of CD11b positive cells with high coexpression of Gr1 (mature granulocytes; 1.8- and 5.6-fold in BM and spleen, resp.) but not low Gr1 coexpression (immature granulocytes) (Figure 1B). Interestingly, this was only seen with the higher dose (20 mg/kg) but not the lower dose (5 mg/kg) of dasatinib.

Both doses of dasatinib significantly increased CD41^+^ megakaryocytic cells in the spleen as well as with 5 mg/kg dasatinib in the BM, while the fraction of these cells was significantly reduced in the BM with 20 mg/kg dasatinib (Figure 1C). The fraction of Ter119^+^ erythroid cells was significantly decreased in BM and spleen after 20 mg/kg dasatinib treatment (Figure 1C).

Spleen weight was slightly, but significantly reduced with the higher but not the lower dose of dasatinib (Figure 1D). Higher doses of dasatinib (20 mg/kg) induced a significant decrease of the fraction of B220^+^ B lymphocytes in the BM and spleen, while lower doses did not (Figure 1E). Furthermore, the fraction of CD4^+^ T cells was significantly reduced in the BM, while CD3^+^ and CD8^+^ cells were not changed (Figure 1E), and there were no changes of CD3^+^, CD4^+^, or CD8^+^ cells in the spleen (Supplementary Table 1A). The ratio between CD4 and CD8 positive CD3^+^ cells was not altered (data not shown). Finally, dasatinib (only 20 mg/kg shown) induced a significant increase of the fraction of NK1.1/CD49b double positive NK cells in the spleen but a decrease in the BM, suggesting mobilization of NK cells from the BM to the spleen (Figure 1F). Analysis of lymphocytes for the expression of CD49b and NK1.1 alone, demonstrated a significant reduction of CD49b^+^ lymphocytes after 20 mg/kg dasatinib in the BM and a similar trend for NK1.1^+^ cells (Supplementary Figure 2A–2B). There was no difference between male and female mice regarding the response to dasatinib (data not shown).

Based on the more pronounced effects of the higher dose of dasatinib (20 mg/kg) and in order to optimize inhibition of the ABL kinase in the BCR-ABL positive mice, we conducted all of the following experiments using this concentration.

### Changes of the immunophenotype after transplantation of BCR-ABL positive bone marrow are reverted by dasatinib treatment of the recipient mice

As different studies showed an immunomodulatory effect of dasatinib in patients with CML and we detected an increase of NK cells in the spleen of wt animals, we tested dasatinib in our CML mouse model.

**Figure 2:**
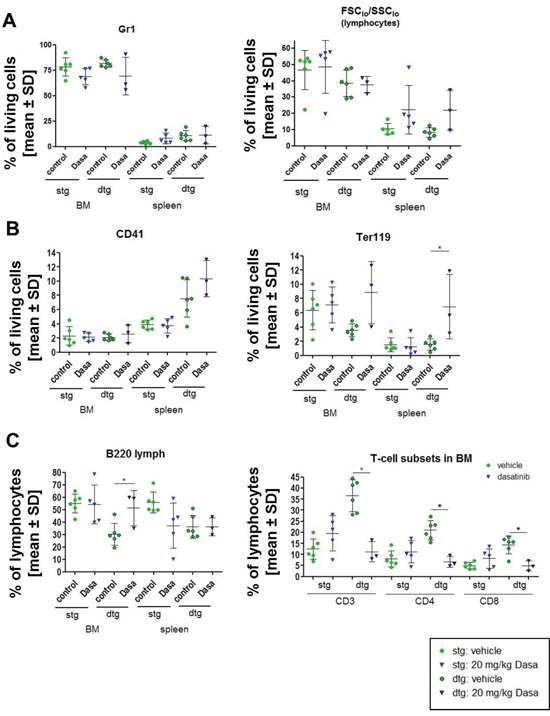
Transplantation of BCR-ABL expressing BM cells influences the immunophenotype of the recipient mice and the dasatinib effect The expression of BCR-ABL was induced in double transgenic (dtg) donor animals and pooled for transplantation of 1×10^6^ BM cells in comparison to BM of SCLtTA single transgenic (stg) control mice. **(A)** After 15 weeks of induction, the mice were treated for 9 days with control vehicle (green circles) or 20 mg/kg dasatinib (blue triangle) (control: stg/dtg: n = 6/6; dasatinib stg/dtg: n = 5/3). Depicted are the percentages of Gr1 positive granulocytic cells and FCS_low_^/^SSC_low_ (lymphocytes) gated on living cells in the BM and the spleen of stg in comparison to dtg mice after dasatinib treatment. **(B)** Analysis of the percentage of CD41 positive megakaryocytes and Ter119 positive erythroid cells in BM and spleen of vehicle and dasatinib treated mice, gated on all living cells. **(C)** Percentage of B220^+^ lymphocytes was evaluated in BM and spleen. Distribution of T cells (CD3, CD4, CD8) in the BM of stg and dtg control or dasatinib treated mice (gated for % of FCS_low_^/^SSC_low_ (lymphocytes)). All data are shown as mean ± SD. *p < 0.05.

For our experimental setup, we induced primary donor FVB/N CD45.1^+^ SCLtTA single transgenic (stg) and SCLtTAxBCR-ABL double transgenic (dtg) animals for 7 days (withdrawal of tetracycline) prior to BM isolation and transplantation into irradiated mice. This transplantation model allows for the detection of cell-intrinsic effects of BCR-ABL positive cells. As expected, immunophenotyping of the pooled bone marrow showed an increase of the fraction of granulocytes in the dtg donor mice (Supplementary Figure 3A). After 9 days of dasatinib or vehicle treatment, we sacrificed the mice for analysis (controls: n=6/group, stg dasa: n=5, dtg dasa: n=3). Due to the transplantation, we detected high percentages of granulocytes in the BM of BCR-ABL positive as well as stg animals (Figure 2A). The fraction of Gr1^+^ cells was increased in the spleen of dtg vs control mice, but there was no change with dasatinib (Figure 2A). The fraction of total lymphocytes in the BM and the spleen of transplanted mice was slightly decreased in BCR-ABL positive mice, but was not significantly affected by dasatinib (Figure 2A). The fraction of CD41^+^ cells (megakaryocytes) in the BM was not altered by either BCR-ABL expression nor affected by dasatinib (Figure 2B). However, in the spleen, the fraction of CD41^+^ cells was more than 2-fold elevated in dtg mice, but this effect was not reversed by dasatinib (Figure 2B). As described before, the fraction of Ter119^+^ erythroid cells was decreased in the BM of BCR-ABL positive mice and reversed by dasatinib (even though this did not reach statistical significance), while there was no significant BCR-ABL induced change of Ter119^+^ cells in the spleen; yet, the fraction of these cells was increased in the spleen of BCR-ABL positive but not control mice (Figure 2B).

The BM of BCR-ABL expressing mice showed a significant decrease of the fraction of B220^+^ B cells, and this effect was reverted by dasatinib (Figure 2C). Interestingly, while the fraction of B220^+^ cells was also reduced in the spleen of BCR-ABL positive mice, dasatinib treatment did not revert this effect in the spleen (Figure 2C).

BCR-ABL positive mice showed a significant increase in the fraction of CD3^+^, CD4^+^, and CD8^+^ T cells in the BM, and this effect was reversed by dasatinib (Figure 2C). No significant differences in the fraction of CD3^+^, CD4^+^, and CD8^+^ T cells were observed in the spleen after dasatinib treatment (Supplementary Table 1B). Analysis of CD49b^+^ lymphocytes showed an increase in the BM of dtg mice that were significantly reduced after dasatinib treatment (Supplementary Figure 3B). In the spleen, no changes were seen in CD49b^+^ lymphocytes (data not shown).

### Dasatinib prolongs survival of transgenic BCR-ABL expressing mice and reverses BCR-ABL induced changes but does not induce lymphocytosis

Since the transplantation procedure used for the previous experiments may have altered some of the dasatinib-induced effects due to the prolonged manifestation of complete hematopoiesis, we additionally assessed the influence of dasatinib treatment under steady-state conditions (without the need for BM transplantation). For these experiments, we induced the expression of BCR-ABL in double transgenic (dtg) mice at day 1 and treated these mice with control vehicle or 20 mg/kg dasatinib starting at day 2.

**Figure 3:**
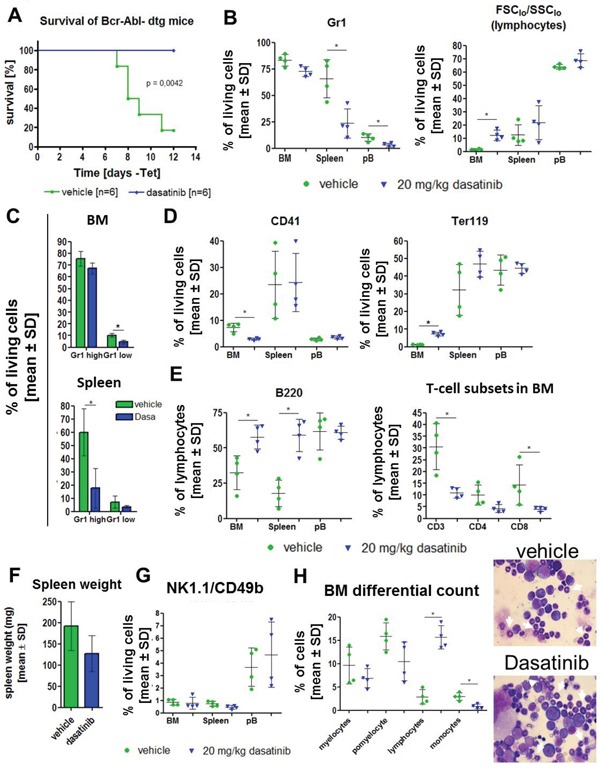
Dasatinib treatment of primary induced mice results in prolonged survival of dtg animals and a reversion of the disease phenotype Expression of BCR-ABL was induced for 1 day by withdrawal of tetracycline from the drinking water of stg (control, BCR-ABL negative) and dtg (BCR-ABL positive) mice. **(A)** Kaplan-Meier survival curve of induced double transgenic (dtg) mice receiving either vehicle control (green) or dasatinb (blue). **(B)** Based on the rapid disease onset in the dtg mice, we induced 8 dtg animals for 1 day and subsequently treated for 9 days with 20 mg/kg dasatinib (blue triangle) or vehicle control (green circle) by oral gavage (n=4/group). Evaluation of granulocytes (Gr1) and lymphocytes (% of FCS_low_^/^SSC_low_) in the bone marrow (BM), spleen and peripheral blood (pB). Depicted are the percentages of living cells. **(C)** Analysis of the amount of mature (Gr1 high/CD11b^+^) and immature (Gr1 low/CD11^+^) granulocytes in the BM and spleen gated on living cells. **(D)** Percentage of megakaryocytes (CD41) and erythroid cells (Ter119) in BM, spleen and pB. **(E)** Analysis of lymphocytes by staining for B220 (B cells) and CD3, CD4 and CD8 (T cells). Shown are the percentages of lymphocytes (FCS_low_^/^SSC_low_). **(F)** Total spleen weight of vehicle treated and dasatinib treated dtg mice. **(G)** Effect of dasatinib on CD49b/NK1.1 double positive NK cells in the BM, spleen and blood. **(H)** Microscopic evaluation of 200 cells in bone marrow smears support the FACS data by showing a decrease of myelocytes, promyelocytes (white arrows) and monocytes and a significant increase in lymphocytes after dasatinib gavage. Shown are two representative Pappenheim stainings from a vehicle treated mouse (upper picture) and a dasatinib treated mouse (lower picture) (100x magnification). All data are shown as mean ± SD. *p < 0.05.

Dasatinib significantly prolonged the survival of BCR-ABL positive (dtg) mice (Figure 3A). Based on these results, we again induced the expression of BCR-ABL directly in primary dtg animals and started the gavage after 1 day without tetracycline with 20 mg/kg dasatinib or vehicle control and analyzed the mice after 7 days of treatment (n=4/group). The last dosing was 1 hour before the analysis. In these BCR-ABL positive mice, dasatinib induced a reduction of the fraction of Gr1^+^ cells in spleen, peripheral blood (PB) (both significant) and BM (non-significant) (Figure 3B). Conversely, there was a dasatinib-induced increase of total lymphocytes (as assessed by FSC_lo_ and SSC_lo_ profile), particularly in the BM (Figure 3B). When analyzing different subsets of Gr1^+^ cells, dasatinib most strongly reduced the fraction of Gr1 high (mature) granulocytic cells in the spleen (with a similar trend in BM), while the effect in Gr1 low (more immature) granulocytes was less pronounced (Figure 3c). As in wild-type animals (Figure 1C), dasatinib reduced the fraction of CD41^+^ cells in the BM (no change in spleen or PB) (Figure 3D). However, different from wild-type mice (Figure 1C), dasatinib increased the fraction of Ter119^+^ cells in the BM (no significant change in spleen or PB) (Figure 3D).

The fraction of B220^+^ B cells was strongly increased in BM and spleen (Figure 3E) as seen in the transplantation setting, both indicative of a reversal of BCR-ABL induced suppression of B cells. The fraction of CD3^+^, CD4^+^, and CD8^+^ cells in BM and spleen was reduced by dasatinib (Figure 3E; changes significant from CD3^+^ and CD8^+^ cells). Analysis of the spleen weight revealed a clinically relevant 34% decrease (Figure 3F), which did not reach statistical significance, possibly due to the short time course of treatment. There was no consistent change of CD49b/NK1.1 double positive NK cells in the presence of dasatinib (Figure 3G), but analysis of CD49b and NK1.1 single positive lymphocytes revealed a significant decrease after dasatinib treatment (Supplementary Figure 4A–4B) that was not seen when gated on all living cells (data not shown). Differential cell counts of the BM confirmed our FACS data by showing a significant increase in lymphocytes and a significant decrease of monocytes, as well as a (non-significant) reduction of myelocytes and promyelocytes (Figure 3H). Single transgenic (stg) animals were treated as a control cohort (n=5 per group) and were analyzed after administration of dasatinib for 11 times, applying the last dose 1 hour before the final analysis (Supplementary Table 3).

### Dasatinib induced changes in the stem and progenitor compartment of BCR-ABL expressing mice

**Figure 4:**
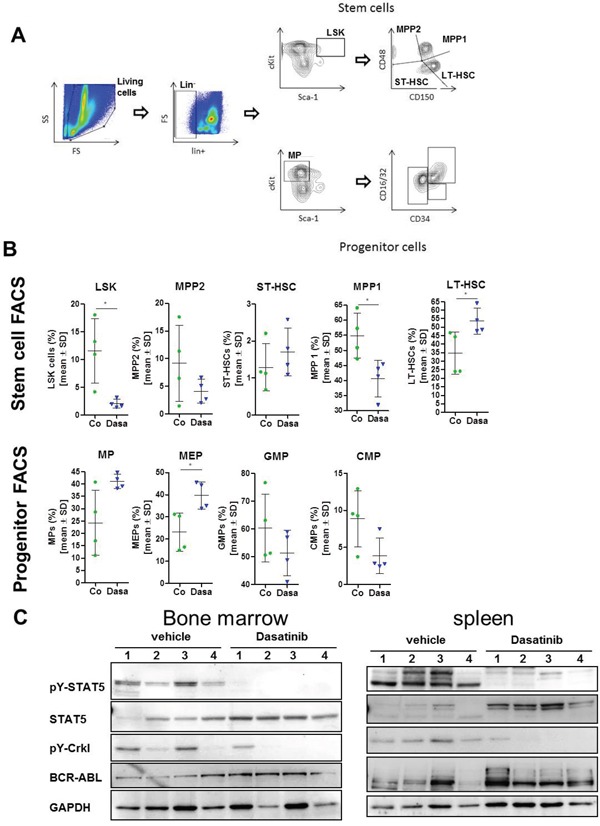
Dasatinib alters the stem- and progenitor cell compartment of BCR-ABL expressing mice After 9 days of treatment with 20 mg/kg dasatinib (blue triangle) or vehicle control (green circle), the stem and progenitor cells in the bone marrow (BM) of BCR-ABL expressing mice were analyzed by FACS staining (n = 4/group) **(A)** Gating strategy for differentiation of stem cells and progenitor cells. **(B)** Stem cells were subdivided into LSK cells (lin^-^, c-kit^+^, Sca-1^+^) that were further characterized for expression of CD48 and CD150. Short-term (ST-, CD48^-^, CD150^-^) and long term (LT-, CD48^-^, CD150^+^) hematopoietic stem cells (HSC) and multi-potent-progenitor: MMP2 (CD48^+^, CD150^-^), MPP1 (CD48^+^, CD150^+^). The progenitor cells were also gated on lin^-^ cells that were analyzed for high expression of c-kit and low Sca-1 expression (MPs). Further differentiation was done by staining for CD34 and CD16/32. MEPs (megakaryocyte-erythroid progenitors) that were CD34 low and CD16/32 positive, while GMP (granulocyte-macrophage progenitor) were positive for both and CMPs (common myeloid progenitor) having lower CD16/32 expression but also high CD34 expression. Depicted are the mean values ± SD. *p < 0.05. **(C)** Western Blot analysis of lysed bone marrow cells as well as spleen cells. Lysates of dasatinib treated mice show less STAT5 and Crkl phosphorylation in comparison to lysates of vehicle treated mice. The indicated antibodies were used for immunostaining. GAPDH served as loading control.

CML is a stem cell disorder with expression of BCR-ABL in various hematopoietic lineages that is mimicked in the SCLtTAxBCR-ABL mouse model. The percentage of Lin^-^Kit^+^Sca-1^+^ (LSK) cells, which are enriched for HSCs, was shown to be increased in induced dtg mice, as well as an expanded GMP (granulocyte-macrophage progenitor) pool, presumably resulting in neutrophilia, as described [17]. We analyzed the stem and progenitor compartment of our dtg animals after 7 days of dasatinib treatment. The gating strategy was adapted from [21] and is depicted in Figure 4A. There was a significant decrease of the fraction of LSK cells as well as the multipotent progenitor-1 (MPP1) population in the dasatinib treated animals (Figure 4B). Interestingly, this was associated with a significant increase in the fraction of phenotypic LT-HSCs (long-term hematopoietic stem cells) compartment, suggesting that these most immature HSCs were spared by dasatinib (Figure 4B). The short term-HSC (ST-HSC) and MPP2 populations were not significantly altered (Figure 4B). The analysis of the myeloid progenitor cells demonstrated a significant increase in the fraction of MEPs (megakaryocyte-erythroid progenitors) by dasatinib, while GMPs and CMPs (common myeloid progenitor) fractions were reduced by dasatinib although this did not reach significance. We confirmed reduction of BCR-ABL kinase activity by Western blotting of lysates generated from unfractionated BM or spleen cells of the dtg mice described in Figure 3, with phosphorylation levels of STAT5 and Crkl being strongly reduced by dasatinib treatment (Figure 4C).

### Histological changes of the lung and intestine after dasatinib treatment

While clearly beneficial for a large proportion of patients with CML, dasatinib treatment has been associated, among other signs and symptoms, with adverse events such as pleural effusions, colitis, or pulmonary hypertension [22]. Interestingly, pleural effusion or colitis were associated with a favorable outcome of the patients with increased cytogenetic and molecular remission [7, 9, 23]. Therefore, we analyzed whether dasatinib treatment was associated with such adverse events or signs for the latter in the mice studied. Gross pathology analysis did not show any clinically relevant pleural effusion.

**Figure 5:**
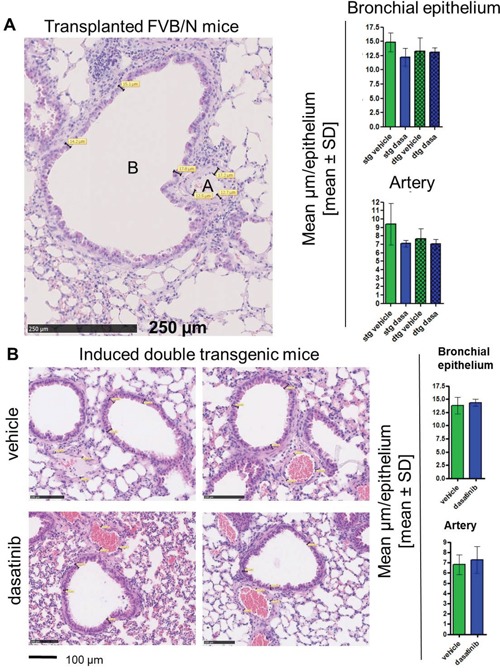
Histological staining and evaluation of the thickness of the lung bronchial epithelium and arteries Analysis of the lung histologies was done on paraffin embedded tissue and hematoxylin and eosin (HE) stained slides. **(A)** Evaluation of the lung vessels in the transplanted FVB/N mice. The size of the bronchial epithelium **(B)** was measured at three different positions, as well as the thickness of the artery walls (A). This measurement was done at three different positions of the lung and the mean value per mouse was calculated. The bar graph shows the mean thickness in μm of the different groups (single transgenic (stg): vehicle n = 6, dasatinib n = 5; double transgenic (dtg): vehicle n = 5, dasatinib n = 3). (B) Representative pictures of the lung histologies of vehicle (upper panel) and dasatinib (lower panel) treated BCR-ABL expressing mice. Scale bar: 100 μm. Four mice per group were analyzed and depicted are the mean values per mouse ± SD of the bronchial epithelium and the artery walls.

Evaluation of lung histologies was performed by measurements of the thickness of muscularis propria of lung arteries and height of bronchus epithelium as depicted in Figure 5. We analyzed three vessels/bronchi per mouse at three different positions and calculated the mean values per lung. In our transplantation model, we saw a slight reduction in thickness in both, muscularis media and bronchial epithelium in the dasatinib treated stg but not dtg mice, however, this did not reach statistical significance (Figure 5A). This was confirmed in the primary transgenic mice (without transplantation) which also showed no significant change in arterial vessels or bronchus epithelia thickness after dasatinib treatment (Figure 5B).

**Figure 6:**
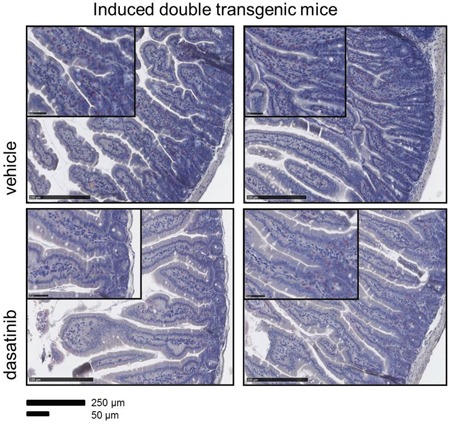
Reduced infiltration of the small intestine by granulocytes in dasatinib treated dtg mice The accumulation of granulocytes in the folds of the small intestine of dtg (double transgenic) mice was demonstrated by immunohistochemistry (NACE staining). Dasatinib treatment of dtg mice strongly reduced the amount of granuloytic infiltration. Scale bar: 250 μm and 50 μm.

There was no dasatinib-induced colitis evident in our mouse model, at least during the short course of dasatinib treatment. In colon mucosa, no changes between the two groups could be seen, but cytochemical NACE staining of the small intestine revealed an infiltration within the epithelium and less in the *lamina propria* by granulocytes in the BCR-ABL expressing mice, which was decreased after treatment with dasatinib (Figure 6).

## DISCUSSION

In this study, we report *in vivo* effects of dasatinib on the cellular composition of the hematopoietic and immune system in wild-type mice as well as in a tetracycline-inducible transgenic mouse model of BCR-ABL positive CML-like disease. We analyzed dasatinib-induced effects by flow cytometry in the bone marrow (BM) and spleen under steady-state conditions as well as after bone marrow transplantation. Furthermore, we conducted flow cytometry analysis of stem- and progenitor cells in the BM of BCR-ABL expressing mice treated with dasatinib or vehicle control and performed histopathology to evaluate effects on the BM, spleen, lung and intestine.

Dasatinib has been previously shown to inhibit neutrophil function *in vitro* and *in vivo* in mice, but effects of dasatinib on neutrophil counts were not reported [24]. We found that 20 mg/kg of dasatinib increased granulocytic cells in normal experimental mice (Figure 1A). Yet, in BCR-ABL positive mice, dasatinib decreased granulocytic cells. This discrepancy may have been due to efficient inhibition of BCR-ABL positive granulocytic cells by dasatinib but failure to induce non-leukemic granulocytes due to insufficient residual normal hematopoiesis. In fact, this is very reminiscent of patients with newly-diagnosed CML, where dasatinib causes neutropenia. As described previously [17], BCR-ABL expression in our transgenic mice induced a decrease of Ter-119 positive erythropoietic cells. Similar to the results in the granulocytic and megakaryocytic lineage, there was a pronounced effect of dasatinib on the BCR-ABL expressing cells, with a recovery of Ter-119 cells in the BM and spleen. This was due to recovered erythropoietic cell production after dasatinib treatment, as shown by the observed MEP increase in the BM (Figure 4B). Previous studies demonstrated a KIT-induced inhibition of the first steps of erythroblast differentiation that can be restored by dasatinib, additionally explaining our findings [25].

Analysis of the lymphocytes of wild-type mice revealed a decrease of B220^+^ B cells upon 20 mg/kg dasatinib treatment, consistent with previous studies that had shown this to be associated with apoptosis of mature B cells and a differentiation block in the immature B cell population [2]. Lower doses may efficiently inhibit B cell development through inhibition of B cell receptor signaling but may not be sufficient to induce apoptosis [26]. B cell depletion in our BCR-ABL expressing mice was reversed by dasatinib, which is in keeping with a BCR-ABL mediated block in B cell differentiation [27] as well as induction of apoptosis of BCR-ABL positive lymphoid cells [28].

In patients, a clonal expansion of cytotoxic T and NK cells was observed during dasatinib treatment that was associated with better therapy response and autoimmune-like adverse reactions [7]. It was hypothesized that the inhibition of immunoregulatory kinases by dasatinib may activate a reversible immune reactivity resulting in good clinical response. These effects were reported to be concurrent with large granular lymphocyte (LGL) lymphocytosis in the majority of cases [10]. This LGL lymphocytosis in the peripheral blood (PB) was not detected in our transgenic mouse model within 7 days of dasatinib treatment (data not shown), but onset of LGL lymphocytosis varied between one and eight months after initiation of dasatinib therapy in patients [10]. FACS analysis of BM and spleen of dasatinib-treated wt animals revealed a reduction of CD4 positive T cells in the BM after 14 days, while the CD3 and CD8 positive cells were not affected. These results are in line with the finding that T cell proliferation was inhibited by dasatinib in a dose-dependent manner in an *in vivo* mixed lymphocyte reaction experiment after adoptive transfer of donor CD4^+^ T cells in allogenic donors [12]. There was a strong increase in the fraction of T cells in the BM and spleen of BCR-ABL mice, both under steady-state conditions and after BM transplantation, which was antagonized by dasatinib treatment (Figure 2C and 3E). Previous studies demonstrated a reduced amount of NK cells, as well as functional defects of NK cells in CML patients at diagnosis [29, 30]. Interestingly, in wt mice, dasatinib led to a decrease of NK1.1/CD49b double positive cells in the BM but an increase in the spleen, suggesting that it enhanced migration of these cells from the BM to the spleen. However, dasatinib treatment of BCR-ABL positive mice did not significantly alter NK cells. With a link between LGL lymphocytosis and cytomegalovirus (CMV) reactivation having been established in CML patients [5], this may not have been mimicked in our BCR-ABL mice due to breeding in isolated ventilated cages. Dasatinib efficiently normalized the LSK, MEP, CMP and GMP fractions in BCR-ABL positive mice with associated normalizations of Gr1^+^ and Ter119^+^ cell populations. However, phenotypic LT-HSCs were spared by dasatinib and their fraction increased. This finding closely reflects reports in CML patients and patient-derived immature CD34^+^CD38^-^ cells that CML stem cells are spared by treatment with TKIs, including dasatinib [28, 31, 32].

To evaluate the occurrence of dasatinib-associated pleural effusion, pulmonary artery hypertension, or colitis, we analyzed the effects on different organs, focusing on the lung and the small intestine, but no evidence for dasatinib induced alterations of the lung or colon were observed. This may have been due to the short duration of dasatinib treatment or to fundamental differences in murine and human immune effects. Nevertheless, dasatinib strongly counteracted BCR-ABL induced granulocytic infiltration of the small intestinal mucosa (Figure 6).

In conclusion, we demonstrate that dasatinib strongly antagonizes BCR-ABL induced effects *in vivo* in a transgenic mouse model of chronic phase-CML, but had differential effects on hematopoiesis in wt mice. Short-term exposure to dasatinib did not induce significant LGL lymphocytosis, pleural effusion or signs of pulmonary artery hypertension. This mouse model is useful to investigate short-term effects of dasatinib and TKI resistance but may also be utilized for assessment of the mechanisms of long-term effects of CML treatment.

## MATERIALS AND METHODS

### Mouse experiments

The SCLtTAxBCR-ABL double transgenic (dtg) mouse model has been described previously [17]. The mice are bred in-house and genotyped with the following primers: tTA forward 5′-GCTAGGTGTAGAGCAGCCTAC-3′ and reverse 5′-GGC GGC ATAC TAT CAG TAG TA-3′ and BCR-ABL forward 5′-GAG CGT GC AGA GTG GAG GGA GAA CA-3′and reverse 5′-GGT ACC AGG AGT GTT TCT CCA GAC TG-3′. In these mice, the expression of BCR-ABL is induced by withdrawal of tetracycline from the drinking water.

Using 8 week old FVB/N wild-type animals purchased from Janvier Labs (France) that were male/female and age matched, two doses of dasatinib were tested, with 5 mg/kg vs. 20 mg/kg dasatinib (provided by Bristol-Myers Squibb) being administered by oral gavage for 14 days. For transplantation experiments, bone marrow (BM) of 4 mice that had been offset from tetracycline for seven days, was pooled and transplanted 1×10^6^ cells (2.5×10^5^ BCR-ABL positive cells; 7.5×10^5^ single-transgenic control cells) into irradiated (10 Gy) FVB/N CD45.2^+^ recipient mice. Afterwards, the disease phenotype was assessed by regular blood counts and flow-cytometric analysis for Gr1/CD11b^+^ cells. After 15 weeks of induction, oral gavage treatment with 20 mg/kg dasatinb or control vehicle (80 mM citric acid stock solution (pH 2.1) in 80 mM citrate buffer (pH 3.1)) was begun and performed for 10 days. The SCLtTAxBCR-ABL mouse strain expresses CD45.1, therefore allowing distinction between donor (CD45.1) and recipient (CD45.2) hematopoiesis.

In another approach BCR-ABL, expression was induced in single-transgenic (stg) control and double-transgenic (dtg) BCR-ABL positive animals, and the mice were treated daily with 20 mg/kg dasatinib for survival analysis. In another cohort of mice, the effect of dasatinb in dtg mice was analyzed after 7 days of treatment.

All animal experiments were approved by the local authorities of North Rhine-Westphalia, Germany (Landesamt für Natur, Umwelt und Verbraucherschutz NRW).

### Dasatinib treatment

Two different batches of dasatinib obtained from Bristol-Myers Squibb (BMS) were used. For the first experiment, dasatinib was dissolved in DMSO, diluted in 80 mM citrate buffer (pH 3.1), and used in comparison with citrate buffer with 5% DMSO (vehicle control). The other batch was prepared as a 30 mg/ml stock solution by sonication in 80 mM citric acid stock solution (pH 2.1). This stock was renewed every five days and diluted daily in 80 mM citrate buffer (pH 3.1) before dosing. Dasatinib was applied once daily with 5 or 20 mg/kg or citrate buffer vehicle by oral gavage. Both batches produced comparable results.

### Flow cytometry analysis

Bone marrow cells were isolated from two tibias and femurs of each mouse by flushing with PBS supplemented with 2% fetal calf serum (FCS). Peripheral blood was obtained by retro-orbital bleeding. Spleen cells were freshly prepared by a 100 μm cell strainer (Greiner Bio-one, Frickenhausen, Germany). Enucleated red blood cells were lysed with ammonium-chloride-potassium buffer (0.15 M NH_4_Cl, 1 mM KHCO_3_, 0.1 mM Na2-EDTA, pH 7.3) prior to incubation with the appropriate FACS antibodies. The following antibodies were used for the phenotyping of the immune cells by FACS analysis: Gr1, CD45.1, B220, CD4, CD11b, CD8, Ter-119, CD3, NK1.1 (Biolegend, Fell, Germany), CD41 (eBioscience, Frankfurt, Germany), CD49b (BD Bioscience, Heidelberg, Germany). 1×10^6^ cells were stained for 15 minutes on ice and washed with PBS/FCS.

For stem- and progenitor cell analysis, cells were stained for lineage positive cells with tricolor- or PE-Cy5 labeled CD4, CD8a, B220 (life technologies, Germany) CD3 (BD Bioscience), Gr1, Ter-119, CD11b (Biolegend). Lineage negative cells were further characterized by CD48, CD150, c-kit (Biolegend) and Sca-1 (biotin labeled first antibody and streptavidin Pe-Cy7 labeled secondary antibody, BD Bioscience) for the stem cell compartment, and CD127, c-kit, CD34 (Biolegend), CD16/32 (BD Bioscience) and Sca-1 for progenitor staining. FACS measurements were conducted with a FACS Gallios (Beckman Coulter, Krefeld, Germany) and analyzed with Kaluza Software (Version 1.3) or FlowJo (Version 10).

### Histology and cytology

The organs were fixed in neutrally buffered 4% formalin before embedding in paraffin. Afterwards, the sections were stained with hematoxylin and eosin (HE) or naphthyl acetate (chloro-)esterase (NACE). Histological figures and quantitative evaluation of lung specimen were done using NPD.view2 software (Hamamatsu) by measuring maximum extend of bronchial epithelia or smooth muscle cells of arterial vessels at three positions in the lung, three measurements each. Blood and bone marrow smears were prepared and Pappenheim stained for the differentiation of the hematopoietic cells (100 cells in the blood smear and 200 in the bone marrow).

### Preparation of cell lysates, SDS-PAGE, and immunoblotting

Cell lysates were prepared, and SDS-PAGE as well as immunoblotting was performed as described before [33]. Proteins on PVDF membrane were detected via chemoluminescence (Fusion SL, PeqLab, Erlangen, Germany). The following antibodies were used: polyclonal rabbit anti-mouse/human phospho-STAT5 (Tyr694, #9351) and phospho-Crkl (Tyr207, #3181) ordered from Cell Signaling/New England Biolabs (Frankfurt, Germany). c-Abl (sc-131), GAPDH (sc-32233) and STAT5 (sc-835) antibodies were purchased from Santa Cruz Biotechnology (Santa Cruz, CA, USA).

### Statistical analysis

GraphPadPrism software was used for the statistical analysis. The two-tailed Student's t-test or Wilcoxon-Mann-Whitney-test were performed, defining significant differences as *P < 0.05, **P < 0.01, ***P < 0.001. Mean and standard deviation (SD) are indicated.

## SUPPLEMENTARY MATERIALS FIGURES AND TABLES



## Abbreviations

BM: bone marrow
CML: chronic myeloid leukemia
CMP: common myeloid progenitor
dtg: double transgenic
GMP: granulocyte-macrophage progenitor
HSC: hematopoietic stem cell
LGL: large granular lymphocytes
LSK: Lin^-^Sca-1^+^Kit^+^
MEP: megakaryocyte-erythroid progenitors
MPP: multipotent progenitor
NK: natural killer cells
PB: peripheral blood
stg: single transgenic
TKI: tyrosine kinase inhibitor
wt: wild-type
