# Supplementary material for: The SCLtTAxBCR-ABL transgenic mouse model closely reflects the differential effects of dasatinib on normal and malignant hematopoiesis in chronic phase-CML patients
Source: Oncotarget. 2017 Mar 13;8(21):34736–49. doi: 10.18632/oncotarget.16152 (PMC5471007; doi:10.18632/oncotarget.16152)
Supplement: Supplementary file 1 [file oncotarget-08-34736-s001.pdf]

The SCLtTAxBCR-ABL transgenic mouse model closely reflects the differential effects of dasatinib on normal and malignant hematopoiesis in chronic phase-CML patients

SUPPLEMENTARY FIGURES AND TABLES

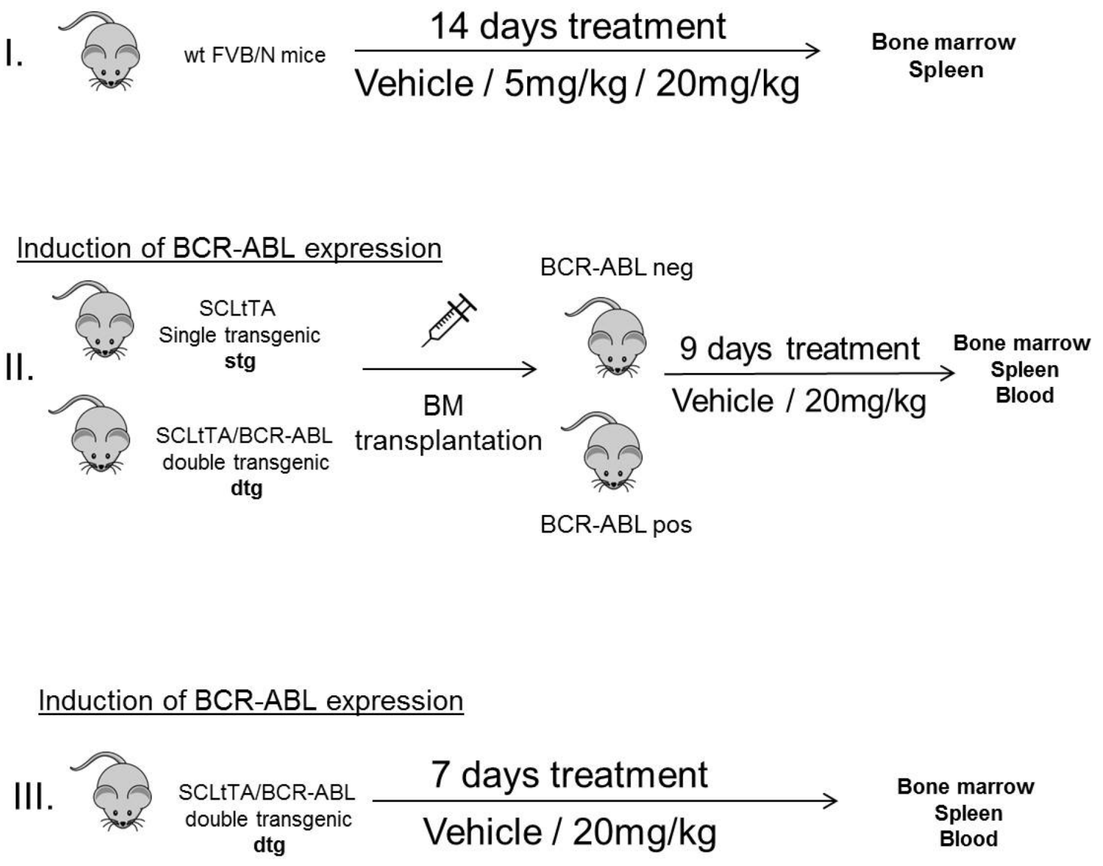

Supplementary Figure 1: Experimental overview. Three independent experiments were conducted. Mice and treatment schedule, as well as the dasatinib concentrations and the analyzed organs are depicted. Stg: single transgenic (BCR-ABL negative); dtg; double transgenic (BCR-ABL positive); BM: Bone marrow.

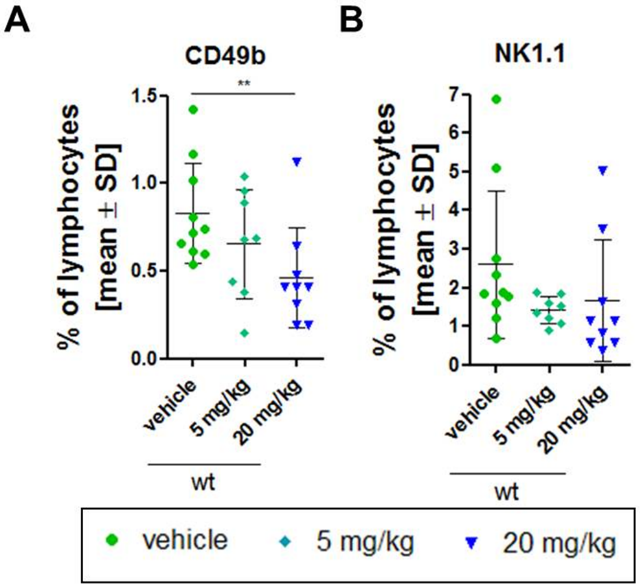

Supplementary Figure 2: Analysis of NK cells in the bone marrow of wt mice. NK cells were stained for the expression of CD49b and NK1.1 **(A)** Expression of CD49b and **(B)** NK1.1 was measured on lymphocytes of wt animals after treatment with vehicle control (n=10; green), 5 mg/kg dastainib (n=8; light blue) and 20 mg/kg (n=9; dark blue). All data are shown as mean ± SD. ***p* < 0.01.

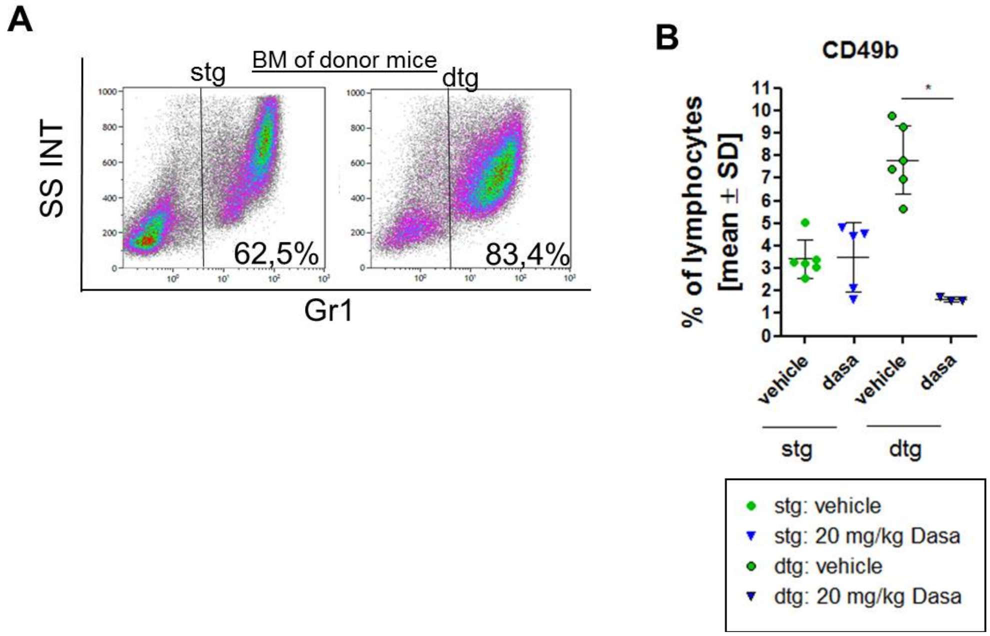

Supplementary Figure 3: FACS analysis of disease onset in stg and dtg mice for transplantation experiments. (A) Single transgenic (stg) and double transgenic (dtg) donor animals were set off tetracycline to induce the expression of BCR-ABL in the dtg mice. Before transplantation of 1x10^6 BM cells into recipient mice, the amount of Gr1 positive cells in the pooled BM of donor mice was analyzed. **(B)** After transplantation of single transgenic (stg) or double transgenic (dtg) BM, the recipient mice were treated for 9 days with control vehicle (green circles) or 20 mg/kg dasatinib (blue triangle) (control: stg/dtg: n = 6/6; dasatinib stg/dtg: n = 5/3). Depicted are the percentages of CD49b positive lymphocytes (FCS_{low}/SSC_{low}). All data are shown as mean ± SD. *p < 0.05.

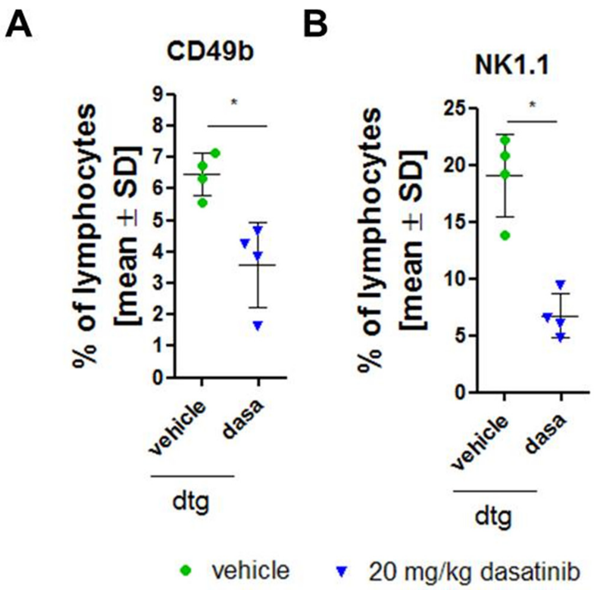

Supplementary Figure 4: FACS plots of CD49b+ and NK1.1+ lymphocytes in the bone marrow of dtg mice. Expression of BCR-ABL was induced for 1 day by withdrawal of tetracycline from the drinking water in dtg (BCR-ABL positive) mice that were treated with either vehicle control (green) or 20 mg/kg dasatinb (blue) (n=4/group). Depicted are the percentages of **(A)** CD49b and **(B)** NK1.1 positive lymphocytes (FCS_{low}/SSC_{low}). All data are shown as mean ± SD. *$p < 0.05$.

Supplementary Table 1: T cell subsets in the spleen

T-cell subsets in the spleen									
(% of lymphocytes: mean % (± SD))									
**A**	**wt**			**B stg transplanted**		**dtg transplanted**		**C dtg**	
	**vehicle**	**5 mg/kg**	**20 mg/kg**	**vehicle**	**20 mg/kg**	**vehicle**	**20 mg/kg**	**vehicle**	**20mg/kg**
	**n=10**	**n=9**	**n=9**	**n=6**	**n=5**	**n=6**	**n=3**	**n=4**	**n=4**
**CD3**	20.6 (± 3.4)	20.8 (± 6.0)	20.7 (± 17.0)	33.8 (± 8.0)	51.9 (± 22.5)	49.2 (± 9.8)	37.7 (± 17.5)	61.5 (± 13.2)	29.8 (± 9.8)*
**CD4**	13.7 (± 3.0)	14.0 (± 5.0)	16.2 (± 12.8)	27.3 (± 6.6)	41.2 (± 20.4)	39.5 (± 7.9)	28.5 (± 16.2)	34.9 (± 12.3)	14.1 (± 6.6)
**CD8**	8.0 (± 0.9)	8.4 (± 1.9)	7.9 (± 6.1)	7.1 (± 1.8)	11.2 (± 2.8)	10.3 (± 2.8)	9.9 (± 1.1)	24.9 (± 5.0)	15.0 (± 3.7)*

FACS analysis of splenic T cells (% of lymphocytes) was done in all experiments. **(A)** CD3, CD4 and CD8 positive lymphocytes were analyzed in wt animals treated with vehicle, 5 mg/kg or 20 mg/kg dasatinib for 14 days. **(B)** After bone marrow transplantation of BCR-ABL expressing or control cells the mice were treated for 9 days with vehicle or 20 mg/kg dasatinib and the T cells in the spleen were analyzed by FACS staining for CD3, CD4 and CD8 positive lymphocytes. **(C)** Percentage of CD3, CD4 and CD8 positive T-lymphocytes in the spleen of BCR-ABL expressing mice after 7 days of 20 mg/kg dasatinib or vehicle treatment. All data are shown as mean ± SD. *$p < 0.05$.

Supplementary Table 2: Analysis of the peripheral blood after transplantation and dasatinib treatment

		peripheral blood			
		mean % (± SD)			
		stg transplanted		dtg transplanted	
		vehicle	20 mg/kg	vehicle	20 mg/kg
		n=6	n=5	n=6	n=3
**% of living cells**	**FSClo/SSClo (lymphocytes)**	10.5 (± 3.3)	22.3 (± 15.0)	8.4 (± 2.9)	21.9 (± 12.4)
	**Gr1**	24.0 (± 13.4)	26.4 (± 24.5)	27.2 (± 14.7)	23.2 (± 18.8)
	**Terr119**	28.4 (± 8.5)	31.1 (± 24.4)	40.3 (± 12.5)	41.7 (± 39.2)
	**CD41**	33.3 (± 6.8)	33.1 (± 8.2)	47.2 (± 8.2)	36.5 (± 10.2)
**% of lymphocytes**	**CD3**	50.1 (± 11.5)	59.6 (± 22.8)	46.4 (± 15.6)	48.3 (± 36.1)
	**CD4**	39.7 (± 9.5)	47.5 (± 18.7)	36.2 (± 13.9)	36.1 (± 26.7)
	**CD8**	10.8 (± 2.7)	12.4 (± 5.1)	10.3 (± 3.2)	12.2 (± 9.3)
	**B220**	18.3 (± 5.0)	11.3 (± 9.3)	11.4 (± 2.3)	8.5 (± 5.2)

After bone marrow transplantation of BCR-ABL expressing or control cells the mice were treated for 9 days with vehicle or 20 mg/kg dasatinib and the peripheral blood was analyzed by FACS for the expression of Gr-1 (granulocytes), Ter119 (erythroid cells) and CD41 (megakaryocytic cells), gated on all living cells. The lymphocytes (FSC_{low}/SSC_{low}) were further characterized by the expression of CD3, CD4 and CD8 (T cells) and B220 (B cells). All data are shown as mean ± SD.
*$p < 0.05$.

Supplementary Table 3 : FACS analysis of single transgenic mice after 11 days of dasatinib treatment

		stg mice after 11 days dasatinib					
		**Bone marrow:**		**Spleen:**		**Blood:**	
		**mean % (± SD)**					
		**vehicle**	**20mg/kg**	**vehicle**	**20mg/kg**	**vehicle**	**20mg/kg**
		**n=5**	**n=5**	**n=5**	**n=5**	**n=5**	**n=5**
**% of living cells**	**FSClo/SSClo (lymphocytes)**	18.1 (± 3.7)	15.0 (2.4)	68.5 (± 3.4)	69.8 (± 2.7)	32.1 (± 12.5)	27.1 (± 5.0)
	**Gr1**	51.6 (± 5.7)	58.3 (± 3.8)	4.3 (± 0.8)	5.5 (± 0.6)	9.6 (± 7.7)	17.8 (± 14.4)
	**Terr119**	13.3 (± 2.8)	13.8 (± 1.9)	53.2 (± 7.5)	53.3 (± 8.0)	18.9 (± 12.4)	24.1 (± 14.4)
	**CD41**	9.3 (± 2.3)	8.2 8± 0.8)	5.2 (± 1.3)	5.0 (± 0.6)	28.7 (± 5.5)	21.4 (± 2.9)
**% of lymphocytes**	**CD3**	5.2 (± 1.8)	9.6 (± 2.4)*	27.1 (± 6.0)	32.1 (± 7.4)*	38.7 (± 19.1)	42.7 (± 14.5)
	**CD4**	2.5 (± 0.7)	4.2 (± 1.1)*	19.0 (± 5.3)	21.5 (± 5.9)*	24.5 (± 14.8)	26.4 (± 11.4)
	**CD8**	2.4 (± 0.7)	4.9 (± 1.2)**	9.3 (± 1.3)	11.4 (± 3.4)**	14.5 (± 4.8)	16.3 (± 4.3)
	**B220**	76.4 (± 7.3)	65.9 (± 6.1)	58.3 (± 6.2)	54.4 (± 7.8)	42.0 (± 15.0)	41.2 (± 13.8)

Bone marrow, spleen and perhipheral blood of single transgenic (stg) mice was analyzed after 11 days of vehicle or 20 mg/kg dasatinib treatment. Percentage of Gr-1 (granulocytes), Ter119 (erythroid cells) and CD41 (megakaryocytic cells), gated on all living cells was analyzed. The lymphocytes (FSC_{low}/SSC_{low}) were further characterized by the expression of CD3, CD4 and CD8 (T cells) and B220 (B cells). All data are shown as mean ± SD. *$p < 0.05$.
